# Genetics of Plasminogen Activator Inhibitor-1 (PAI-1) in a Ghanaian Population

**DOI:** 10.1371/journal.pone.0136379

**Published:** 2015-08-31

**Authors:** Marquitta J. White, Nuri M. Kodaman, Reed H. Harder, Folkert W. Asselbergs, Douglas E. Vaughan, Nancy J. Brown, Jason H. Moore, Scott M. Williams

**Affiliations:** 1 Center for Human Genetics Research, Vanderbilt University, Nashville, Tennessee, United States of America; 2 Department of Genetics and Institute of Quantitative Biomedical Sciences, Dartmouth College, Hanover, New Hampshire, United States of America; 3 Department Heart & Lungs, University Medical Center Utrecht, Heidelberglaan 100, 3584CX, Utrecht, the Netherlands; 4 Institute of Cardiovascular Science, University College London, 222 Euston Road, London, United Kingdom; 5 Durrer Center for Cardiogenetic Research, ICIN-Netherlands Heart Institute, Utrecht, The Netherlands; 6 Department of Medicine, Northwestern University, Feinberg School of Medicine, Chicago, Illinois, United States of America; 7 Department of Medicine Vanderbilt University, Nashville, Tennessee, United States of America; Sant Joan de Déu Children's Hospital, SPAIN

## Abstract

Plasminogen activator inhibitor 1 (PAI-1), a major modulator of the fibrinolytic system, is an important factor in cardiovascular disease (CVD) susceptibility and severity. PAI-1 is highly heritable, but the few genes associated with it explain only a small portion of its variation. Studies of PAI-1 typically employ linear regression to estimate the effects of genetic variants on PAI-1 levels, but PAI-1 is not normally distributed, even after transformation. Therefore, alternative statistical methods may provide greater power to identify important genetic variants. Additionally, most genetic studies of PAI-1 have been performed on populations of European descent, limiting the generalizability of their results. We analyzed >30,000 variants for association with PAI-1 in a Ghanaian population, using median regression, a non-parametric alternative to linear regression. Three variants associated with median PAI-1, the most significant of which was in the gene *arylsulfatase B* (*ARSB*) (p = 1.09 x 10^−7^). We also analyzed the upper quartile of PAI-1, the most clinically relevant part of the distribution, and found 19 SNPs significantly associated in this quartile. Of note an association was found in *period circadian clock 3* (*PER3*). Our results reveal novel associations with median and elevated PAI-1 in an understudied population. The lack of overlap between the two analyses indicates that the genetic effects on PAI-1 are not uniform across its distribution. They also provide evidence of the generalizability of the circadian pathway’s effect on PAI-1, as a recent meta-analysis performed in Caucasian populations identified another circadian clock gene (*ARNTL*).

## Introduction

Cardiovascular disease (CVD) consists of multiple conditions with overlapping environmental and genetic risk factors, symptoms, and disease etiologies. It causes ~48% of all non-communicable disease-related deaths worldwide[1]. Thrombosis is a major factor in CVD, including myocardial infarction (MI) and stroke, representing an excellent target for CVD prevention and treatment[2]. Fibrinolysis, the process by which the clotting protein fibrin is cleaved by plasmin, moderates thrombotic events[3]. Impairment of the fibrinolytic balance is due in part to increased plasminogen activator inhibitor-1 (PAI-1) and associates with thrombotic risk and severity [4]. Although several studies of plasma PAI-1 levels indicate a positive correlation with susceptibility to thromboembolism, atherosclerosis, and MI, the nature of the relationship between PAI-1 and CVD risk remains inadequately defined[5,6].

PAI-1 levels are influenced by genetic variation, with heritability estimates ranging between 0.42–0.71[7–9]. The most studied genetic variant, impacting PAI-1 levels, is the 4G/5G promoter polymorphism. This variant influences circulating PAI-1 levels in a dose-dependent manner, with carriers of the 4G allele exhibiting higher levels of circulating PAI-1[10]. However, this variant alone does not account for most of the PAI-1’s heritability. Other variants must also play a significant role in the variation of PAI-1 levels[10]. The majority of studies aimed at uncovering these variants have been conducted in Caucasian populations [11]. Few studies have been performed on African populations, most notably using a population-based cohort form the Brong Ahafo region in Sunyani, Ghana[12,13]. These African-based studies, however, were candidate gene analyses focusing on a relatively small number of nucleotide polymorphisms (SNPs). A small number of other studies have investigated racial/ethnic group differences in mean PAI-1 levels [14–17]. While the results from these reports are highly variable, the non-normal nature of the PAI-1 distribution is a highly conserved feature, regardless of racial/ethnic group.

A possibly limiting factor in elucidating the genetic bases of PAI-1 has been the use of linear regression to determine the association of SNPs with PAI-1 levels. This may not be an appropriate analytical method as PAI-1 is highly skewed[18]. Furthermore, using the mean to assess the effect of SNPs on PAI-1 in the presence of extreme values can also lead to incorrect inference[19]. Therefore, the median of the distribution may be a more appropriate measure of the overall behavior of PAI-1 levels in response to genotype. Quantile regression is a non-parametric alternative to linear regression that parallels standard linear regression conceptually, but makes no assumptions about the distribution of residual errors, and is therefore robust to skewness and heteroskedasticity in the phenotypic distribution [18,20,21]. Quantile regression can be used to assess the impact of any percentile of the phenotypic distribution. We applied quantile regression to assess the effect of genotype on the median of the PAI-1 distribution.

Both median regression and standard linear regression assume uniform effects of the independent variable on the dependent variable, for which there is no theoretical justification or empirical support in the case of PAI-1. Recent evidence from genetic studies of other biological traits raises the possibility that genetic variants exhibit quantile-specific effects [19,22], but this has been ignored in most genetic studies and all previous studies of PAI-1. Quantile regression can be used to estimate the effects of specific genetic variants in different parts of the phenotypic distribution[19]. This may be of special interest when specific ranges of a distribution relate to a particular clinical endpoint. Since elevated PAI-1 levels are associated with CVD, the impact of genetic variants in different quantiles of the PAI-1 distribution may be of special clinical interest. Quantile regression has been used sparingly in human genetics studies although it has been used more extensively other fields[19]. We explicitly tested the hypothesis that there are genetic factors with non-uniform and/or non-linear effects on plasma PAI-1 by performing quantile regression on the 75^th^ percentile of PAI-1 values.

## Methods

### Study Cohort Description

All participants provided written consent for this study. The study comprised a subset of 1105 unrelated individuals recruited from Sunyani, Ghana recruited between 2002 and 2005 [23]. We examined a subset of the cohort previously assessed [12,13] (n = 992) and an additional 113 subjects from the 90th percentile of the plasma PAI-1 distribution. Ascertainment, DNA collection and biomarker measurement protocols are described elsewhere[23]. DNA and demographic data including age, body mass index (BMI), triglycerides and PAI-1 levels, were collected for all study participants. This study was approved by the Committee for the Protection of Human Subjects at Dartmouth College.

### Genotyping Scheme

DNA was genotyped using the Illumina Infinium HumanExome BeadChip (Exome Chip) platform (Illumina, Inc., San Diego, CA). The Exome Chip provides coverage of the exonic regions of the genome, using approximately 240,000 markers. We supplemented these with an additional 8,439 common variants selected to target genes with prior evidence of association with variation in CVD.

### Quality Control Procedures

Participants with genotyping efficiency less than 95% were excluded from further analysis. Subjects who were missing demographic and/or biomarker data were also excluded from the study. After quality control (QC) procedures, 1053 individuals (441 males, 612 females) remained. A total of 39,124 common variants (minor allele frequency ≥ 0.05) were analyzed from the Exome Chip genotype data. QC criteria for the selection of common variants included a genotyping efficiency > 95% and a Hardy-Weinberg equilibrium p-value > 0.001, after which 38,871 variants remained. All QC was carried out with PLINK[24].

### Preliminary Analyses

The distributions of demographic and biological variables were assessed in males and females, separately, to determine if any significant differences existed between sexes. Normality of continuous traits was evaluated using the Shapiro-Wilk test (p < 0.05). For normally distributed continuous variables, the Student’s t test was used to assess mean differences between sexes. In cases of non-normality, the Wilcoxon rank sum test was used. For discrete variables, the Chi-square test was used. Analyses were performed using STATA 11[25].

To be included in the study, both parents and both sets of grandparents had to be native to Ghana, reducing likelihood of population stratification. However, as an added precaution, we explicitly tested for substructure using STRUCTURE[26]. This analysis was performed using 8521 variants pruned for LD (r^2^ = 0.5) and present in the JPT+CHB, YRI, and CEU HapMap data. STRUCTURE runs used an admixture model with correlated allele frequencies (burn-ins = 10,000; iterations = 10,000) in a supervised analysis with K = 3. STRUCTURE analysis revealed no significant evidence of population stratification within our dataset (S1 Fig).

### Median Regression Analysis

The distribution of PAI-1 levels was tested for normality and found to deviate even after log transformation (Shapiro-Wilk test p < 0.001). Therefore, median regression was performed with the quantreg package in R[27]. Regression models were adjusted for age, sex, BMI, triglycerides, and genotype at the *PAI-1* 4G/5G variant. Triglyceride levels were log-transformed. Single variant results were visualized using the qqplots package in R [28]. Because a large number of the variants genotyped were in moderate to high LD, Bonferroni correction was deemed overly conservative. Therefore, False Discovery Rate (FDR) was also used to correct for multiple testing. FDR is more robust to the violation of the independent test assumption, providing moderate control of type I error. Results were considered statistically significant if p < 2.57x10^-6^ (FDR q = 0.1).

To test if median regression exhibited greater sensitivity to detect linear and non-linear effects than linear regression, we constructed linear regression models for SNPs found to be significant with median regression. Linear regression models were adjusted for the covariates above. Analyses were performed with STATA 11[25].

Additive models were used, with the major allele as referent. In cases where there were fewer than five individuals in a genotype group, SNPs were coded dominantly for the effect of the minor allele, i.e., the homozygous minor and heterozygote genotype groups were combined into one class and compared to the homozygous major genotype.

### Exploratory Upper PAI-1 Quartile Regression

Because the upper extremes of the PAI-1 distribution associate with clinical outcomes, we performed upper quartile regression to assess the impact of single variants within this target region of the PAI-1 distribution [29,30]. The quantreg package in R was used [28], with the option for robust standard errors. SNPs were coded as described above. For gene regions contained more than one associating variant, pairwise LD was assessed using Haploview [31].

### Bioinformatic / Data Mining Investigation of Associating markers

We used the Function SNP Prediction (FuncPred) pipeline in SNPnfo [32] that incorporates several software tools/web servers such as PolyPhen [33], SNPs3D[34], MATCH[35], and ESEfinder [36] to assess the possibility that SNPs affect biological function (i.e. protein structure/stability, exon splicing, transcription, etc.).

## Results

### Cohort Demographics

There were no significant differences in mean age, triglyceride levels, plasma PAI-1 levels, or distribution of PAI-1 4G/5G variant genotypes between sexes (Table 1). Females had higher BMIs than males (p < 0.001).

**Table 1:** HeART cohort gender-separated demographics.

		Males (n = 441)	Females (n = 612)	P-Value^a.^
**Age (years)**		44.02(12.47)	43.22(10.75)	0.523
**Body Mass Index**		24.21(4.29)	27.08(5.44)	<0.001
**Triglycerides**		94.45(52.85)	93.89(56.24)	0.489
**serum PAI-1 levels**		7.96(8.90)	8.84(11.02)	0.930
**PAI-1 4G/5G**	**4G/4G**	20	34	0.264^b^
	**4G/5G**	108	171	
	**5G/5G**	260	332	

^a.^ P-values are from the Wilcoxon Rank Sum test unless otherwise indicated.

^b.^ P-values are derived from the Chi-square test of association.

### Median Regression Analyses

**Fig 1:**
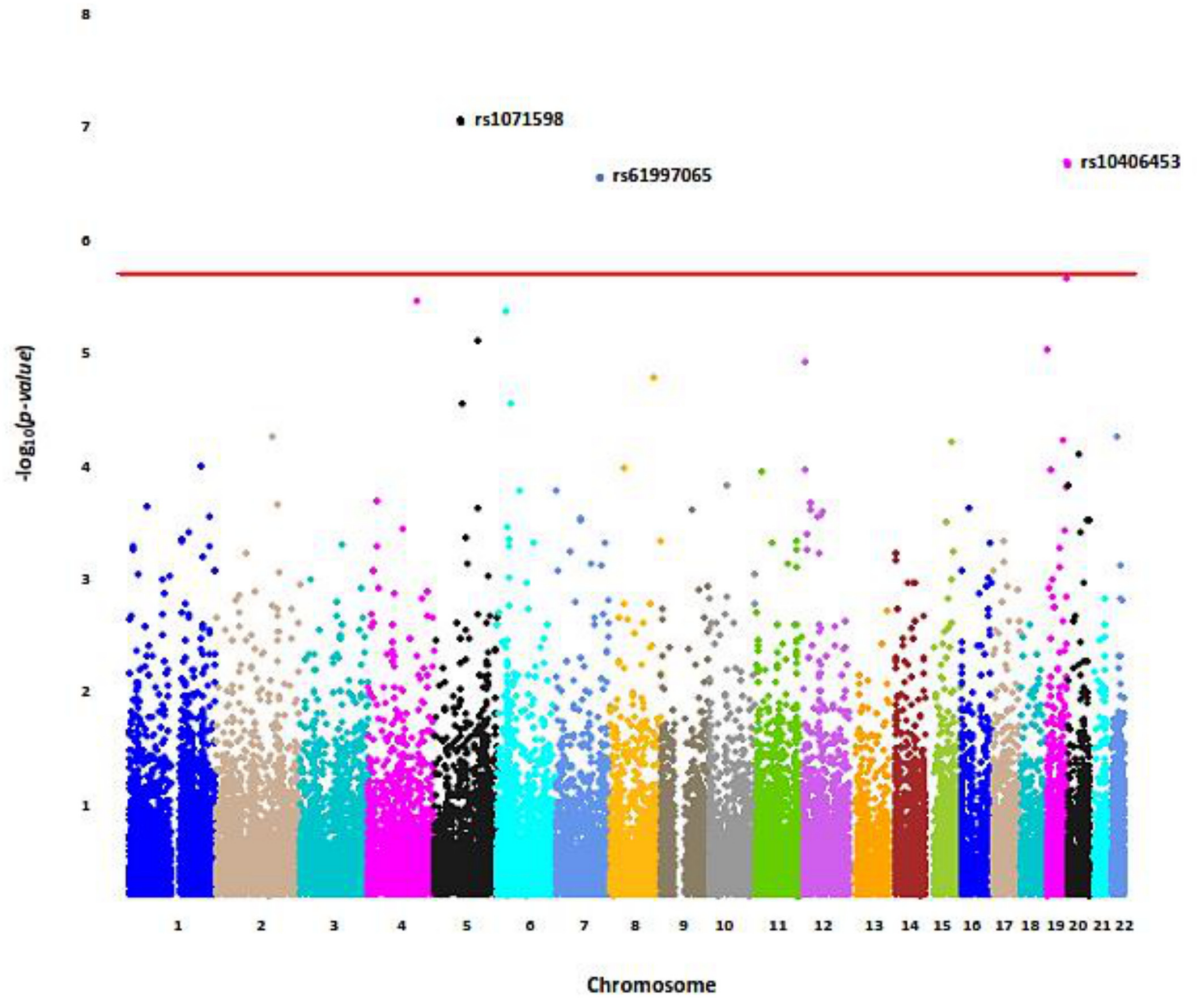
Manhattan Plot of Common Variant Association Analysis with Median plasma PAI-1. Only markers on chromosomes 1–22 are presented above; regions from the X chromosome, Y chromosome, pseudo-autosomal region of the X chromosome, and mitochondrial markers have been excluded. Statistically significant markers are labeled in bold. Red Line represents FDR significance threshold (2.57 x 10^−6^).

Three non-synonymous single nucleotide polymorphisms (SNPs) significantly associated with circulating PAI-1 levels after Bonferroni correction. These were SNPs rs1071598 (p = 1.09 x 10^−7^), rs61997065 (p = 3.56 x 10^−7^), and rs10406453 (p = 2.58 x 10^−7^), located on chromosomes 5, 7, and 19, respectively (Table 2, Fig 1). Of these variants, rs1071598, a missense SNP located in the *arylsulfatase B* (*ARSB)* gene, and rs10406543, a missense variant in *leukocyte receptor cluster member 9* (*LENG9)* had similar effects on median PAI-1 levels (rs1071598 β = -0.442, rs10406543 β = -0.467; where β represents the allelic effect on the natural log transformed PAI-1 level.) (Table 2). In contrast, rs61997065, in *carboxypeptidase A2* (*CPA2)*, displayed a strong positive effect on median PAI-1 levels (β = 0.503) (Table 2).

**Table 2:** Median Regression Results for Single Variant association with circulating Plasminogen Activator Inhibitor 1 (PAI1) levels. Results that remained significant after FDR correction are highlighted in **bold**; only a subset of significant results are presented above (p-value ≤ 10^−5^).

Chr.	Gene	SNP	Beta^a.^	SE^b.^	95% Confidence Interval		P-value
					LL	UL	
4	*SLC7A11*	rs4479754	-0.293	0.064	-0.418	-0.168	4.67E-06
5	*ARSB*	rs1071598	-0.442	0.083	-0.604	-0.280	**1.09E-07** ^‡^
7	*CPA2*	rs61997065	0.503	0.098	0.311	0.695	**3.56E-07** ^‡^
19	*LENG9*	rs10406453	-0.467	0.090	-0.643	-0.290	**2.58E-07** ^‡^
19	*LENG8*	rs1035451	-0.375	0.080	-0.532	-0.219	2.90E-06
7	*SERPINE1*	rs1799768 (PAI-1 4G/5G)	0.266	0.071	0.123	0.405	1.84E-04

^a.^Beta coefficient from median regression model represents the effect of the minor allele; model covariates: age, sex, BMI, triglycerides, and PAI1 4G/5G variant genotype.

^b.^SE; Standard error; robust standard errors are reported above. LL = 95% Confidence Interval lower limit; UL = 95% Confidence Interval upper limit

^‡^Effect remained significant after Bonferroni correction (threshold = 1.29E-06)

We tested the five significant or marginally significant SNPs (p < 10^−5^) using standard linear regression models adjusted for the same covariates as above (S1 Table). For each SNP, the effect trended in the same direction; however, in every model, the standard error reported by linear regression was greater than that reported by median regression. This resulted in larger 95% confidence intervals and larger p-values, confirming that median regression in a skewed data set can increase sensitivity (S1 Table).

We also evaluated the effect of the PAI1 4G/5G promoter polymorphism (rs1799768), shown in multiple populations to have a strong impact on PAI-1 levels [11,13], using both median regression and standard linear regression (Table 2, S1 Table). Association results from the two regression methods were comparable (linear regression β = 0.25; p = 1.30 x 10^−4^ and median regression β = 0.27; p = 1.84 x 10^−4^).

### Quartile Regression Analyses

**Fig 2:**
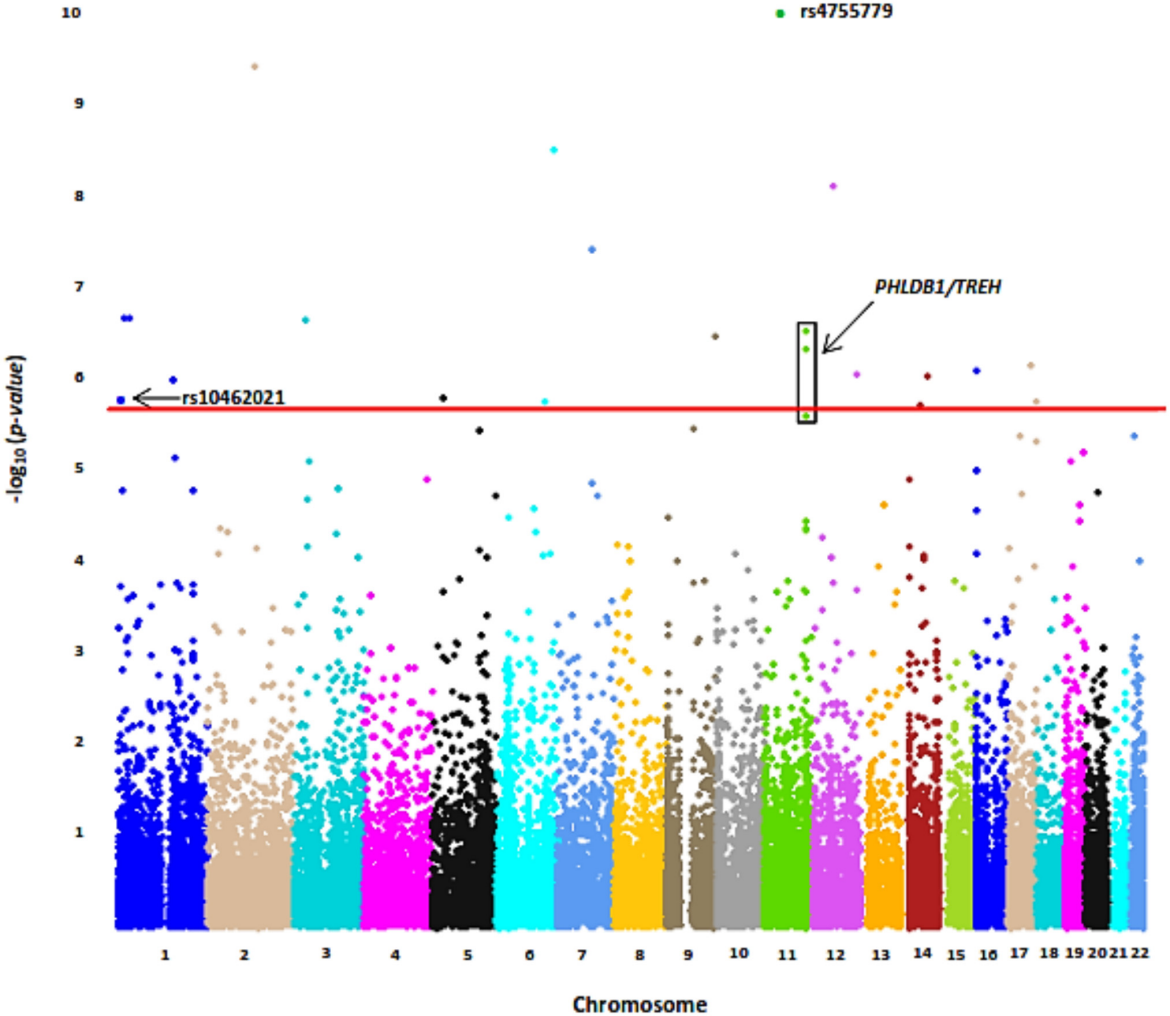
Manhattan Plot of Common Variant Association Analysis with Upper Quartile of PAI-1 distribution. Only markers on chromosomes 1–22 are presented above; regions from the X chromosome, Y chromosome, pseudo-autosomal region of the X chromosome, and mitochondrial markers have been excluded. Top three significant markers are labeled in bold. Red Line represents FDR significance threshold (2.57 x 10^−6^). Loci with more than one significant variant are signified by a box. Noteworthy significant associations are labeled in bold.

Quantile regression analyses were performed on the upper quartile of the PAI-1 distribution. Nineteen variants were significant after correction for multiple testing with FDR (Table 3, Fig 2). The most significant effect in the upper quartile of the PAI-1 distribution was observed for rs4755779, located on chromosome 11 (p = 1.44 x 10^−10^), while the largest negative and positive effects were observed for rs10462021 (β = -0.434), located on chromosome 1, and rs116307792 (β = 0.249), located on chromosome 3 (Table 3). Of note, a 72.6kb region on chromosome 11 containing both the *pleckstrin homology-like domain*, *family B*, *member 1 (PHLDB1*) and *trehalase (TREH)* genes (*PHLDB1/TREH* gene region) harbored three SNPS that were significantly associated in the upper quartile, two of which (rs7389 and rs519982) remained significant after Bonferroni correction.

**Table 3:** Upper Quartile Regression Results for Single Variant association with circulating Plasminogen Activator Inhibitor 1 (PAI-1) levels. Results that remained significant after FDR correction are highlighted in **bold**; only a subset of significant results are presented above (p-value ≤ 10^−6^).

Chr.	Gene	SNP	Beta^a.^	SE^b.^	95% Confidence Interval		P-value
					LL	UL	
1	*COL16A1*	rs72887331	-0.277	0.053	-0.381	-0.172	**2.64E-07** ^‡^
1	*FHAD1*	rs12126178	-0.133	0.026	-0.184	-0.083	**2.64E-07** ^‡^
1	*PER3*	rs10462021	-0.434	0.091	-0.613	-0.256	**2.07E-06**
2	*PLEKHB2*	rs6713972	-0.234	0.037	-0.308	-0.161	**5.14E-10** ^‡^
3	—	rs13314993	0.212	0.041	0.132	0.293	**2.85E-07** ^‡^
3	—	rs33483	0.202	0.045	0.113	0.291	9.48E-06
3	*SLC15A2*	rs116307792	0.249	0.047	0.158	0.340	**1.08E-07** ^‡^
5	*ADAMTS12*	rs61757473	-0.375	0.063	-0.500	-0.252	**3.26E-09** ^‡^
5	*RAPGEF6*	rs61757473	-0.178	0.039	-0.254	-0.103	4.43E-06
6	*TAGAP*	rs35263580	-0.198	0.033	-0.263	-0.133	**4.14E-09** ^‡^
7	—	rs2023783	-0.432	0.079	-0.586	-0.278	**4.97E-08** ^‡^
9	*DBH*	rs4531	-0.195	0.038	-0.270	-0.120	**4.19E-07** ^‡^
9	*NMRK1*	rs35472028	-0.274	0.059	-0.391	-0.158	4.19E-06
11	*EXT2*	rs4755779	-0.213	0.033	-0.277	-0.148	**1.44E-10** ^‡^
	*PHLDB1 / TREH*	rs7389	-0.252	0.049	-0.349	-0.155	**3.70E-07** ^‡^
11	*PHLDB1 / TREH*	rs2077173	-0.256	0.055	-0.363	-0.149	3.05E-06
	*TREH*	rs519982	-0.259	0.051	-0.360	-0.158	**5.75E-07** ^‡^
12	*OR1OP1*	rs76940436	-0.268	0.046	-0.359	-0.177	**1.00E-08** ^‡^
12	*P2RX7*	rs34219304	-0.302	0.062	-0.423	-0.181	**1.09E-06** ^‡^
14	*FAM161B*	rs34834232	-0.258	0.053	-0.361	-0.155	**1.13E-06** ^‡^
14	*NID2*	rs2273430	-0.239	0.050	-0.338	-0.141	**2.32E-06**
16	*C1QTNF8*	rs73494080	-0.283	0.057	-0.395	-0.170	**9.82E-07** ^‡^
17	—	rs4796217	-0.211	0.046	-0.301	-0.121	4.94E-06
17	*CEP95*	rs9910506	-0.327	0.066	-0.457	-0.198	**8.70E-07** ^‡^
17	*SECTM1*	rs113432525	-0.276	0.060	-0.394	-0.157	5.63E-06
19	*ERVV-1*	rs10403404	-0.201	0.045	-0.289	-0.114	7.60E-06
19	*PDE4C*	rs1444689	0.224	0.050	0.125	0.322	9.16E-06
22	*C22orf43*	rs75824255	0.185	0.040	0.106	0.263	4.92E-06

^a.^Beta coefficient from median regression model represents the effect of the minor allele; model covariates: age, sex, BMI, triglycerides, and PAI1 4G/5G variant genotype.

^b.^SE; Standard error; robust standard errors are reported above. LL = 95% Confidence Interval lower limit; UL = 95% Confidence Interval upper limit

^‡^Effect remained significant after Bonferroni correction (threshold = 1.29E-06)

## Discussion

Susceptibility to major thrombotic events is increased by unbalanced or impaired fibrinolysis, which is heavily impacted by variation in PAI-1 levels. Our results identified three novel variants that significantly associated with median PAI-1. We further postulated that the effects of genetic variants on PAI-1 were non-uniform across its distribution, and tested this hypothesis by investigating the impact of common variants on the clinically relevant upper quartile. We found 19 SNPs that were significantly associated with PAI-1 levels in the upper quartile, including one region that harbored multiple associating variants. Our study not only revealed novel associations with PAI-1 levels but also found the first evidence for association in an African population of quartile-specific effects on PAI-1 levels.

### Median regression

Of the three SNPs that associated with median PAI-1, rs1071598 was the most significant. Located within the fourth exon of *ARSB*, rs1071518 is responsible for a valine to methionine amino acid change at position 376 (V376M) that is classified as “probably benign” with respect to its effect on ARSB protein function[32]. Although there is no strong evidence that this SNP affects ARSB protein function, the V376M substitution may affect structural stability. Methionine to valine substitutions are predicted to cause over-packing of protein cores, as methionine is a larger amino acid than valine, possibly influencing protein stability[37]. According to SNPinfo, rs1071598 is located within two base pairs of a putative exon splice enhancer motif, potentially affecting the relative frequency of splice variants. *ARSB* has been implicated in reactive oxidative species (ROS) production and the activation of ROS-mediated inflammatory cascades [38]. ARSB also has the ability both to replicate and mediate the effects of hypoxia in human tissue [39]. *PAI-1* was recently identified as a hypoxia inducible gene, and has long been established as a biomarker of inflammation [40]. The shared connection with inflammatory responses of *ARSB* and *PAI-1* presents a potential link between PAI-1 levels and genetic variants in *ARSB*.

Another SNP, rs61997065, located in the only exon of *LENG9*, has an effect similar to the *ARSB* SNP. It causes a histidine to arginine substitution (H153R) predicted to be benign with respect to protein function. *LENG9* is a member of the *leukocyte receptor complex* (*LRC*), an extended gene region on chromosome 19 that encodes immunoglobulin superfamily receptors [41]. Although *LENG9* has been mapped to the *LRC*, its function is unknown.

The only SNP that associated with increased PAI-1 levels was rs61997065, located in *CPA2*, which causes a valine to isoleucine substitution (V67I). This SNP is proximal to a predicted exon splice enhancer motif, indicating a possible biological role[32]. *CPA2* is a digestive exopeptidase found primarily in the pancreas that is also expressed in the brain, in both humans and rats [42,43]. Previous studies revealed a possible regulatory role of extrapancreatic *CPA2* in the renin-angiotensin system (RAS) via differential processing of Angiotensin I [44,45]. There are multiple sources linking the RAS and the fibrinolytic system [46,47]. Additionally, genetic variants of the RAS have been previously associated with mean PAI-1 levels in both Caucasian and African populations [12,48].

### Upper quartile regression

Upper quartile regression analyses identified 19 associating variants; of particular note among these variants were 1) two non-synonymous SNPs located in genes with a plausible connection to PAI-1, rs4755779 in *EXT2* and rs10462021 in *PER3*, and 2) three SNPs located in the *PHLBD1/TREH* gene region on chromosome 11.

The *EXT2* SNP, rs4755779, is a missense variant that causes a methionine to valine substitution (M42V), predicted to be benign with respect to protein function. *EXT2* encodes a protein involved in heparin sulfate biosynthesis, and associates with hereditary multiple exostoses and type 2 diabetes [49,50]. A plausible biological connection exists between *EXT2* and *PAI-1* via heparin-binding growth factors (HBGF). HBGFs have been implicated in the modulation of *PAI-1* expression. In particular, HBGF-1 inhibits *PAI-1* expression in human umbilical vein endothelial cells [51].

**Fig 3:**
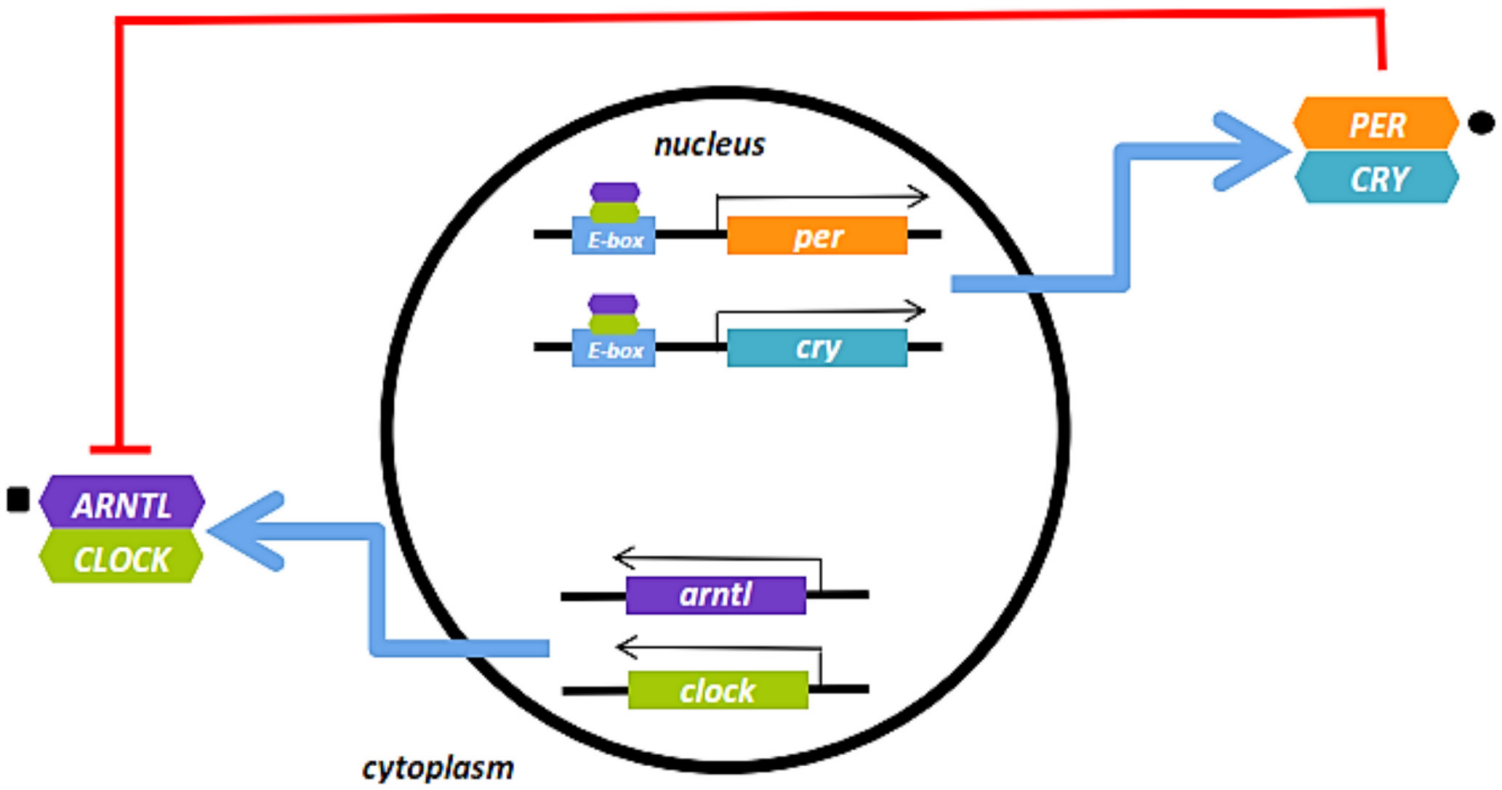
Schematic of the Circadian Rhythm Pathway. Figure above was adapted from Stow *et al*. 2011[57]. Square indicates a significant association with mean PAI-1 levels in Caucasian populations; circle indicates a significant association with PAI-1 levels in the current study.

An associating missense variant in PER3, rs10462021, is responsible for a histidine to arginine substitution (H1139R), and is predicted to have an effect on protein function, although the nature of this effect is unclear. *PER3* is a member of the circadian rhythm pathway that affects inflammatory responses by increasing the secretion of pro-inflammatory cytokines [52]. Previous studies in model organisms have also reported an association between *PER3* and susceptibility to CVD, and transgenic *PER3* knockout mice showed increased susceptibility to arteriosclerotic disease[53]. The identification of rs10462021 in *PER3* is particularly noteworthy because variants in another prominent member of the circadian rhythm pathway, *aryl hydrocarbon receptor nuclear translocator-like gene* (*ARNTL*), were found to be associated with *PAI-1* levels in a recent meta-analysis performed on Caucasians [11]. PER3 and ARNTL are major regulators of the circadian clock mechanism, a transcriptional timing apparatus governed by multiple positive and negative feedback loops[54]. ARNTL forms a heterodimer with CLOCK, which drives transcription of the *PER* and *CRY* gene families. PER and CRY then heterodimerize to form a complex that acts as an inhibitor of the ARNTL/CLOCK complex, creating a negative feedback loop[54] (Fig 3). The interaction between the PER3/CRY and ARTNL/CLOCK heterodimers is of note because there is substantial evidence that ARNTL/CLOCK activates the *PAI-1* promoter and increases *PAI-1* expression [55,56].

The effects of *PER3* and *ARNTL* on PAI-1 variation may be population specific, but the involvement of the circadian rhythm pathway appears to be generalizable. A difference in allele and genotype frequencies at the *PER3* variant, rs10462021, may be responsible, in part, for a population-specific effect as found in a study comparing world-wide populations[58]. Allele frequency differences between African and European descent populations may affect the ability to detect or replicate the effects of this variant. However, the fact that multiple circadian clock genes have been associated with PAI-1 indicates the importance of the pathway despite possibly variable effects of specific genes.

We also discovered a 72.6kb region on chromosome 11, containing two genes, *PHLBDI* and *TREH*, with multiple associating variants. Of the three variants identified in this region, two (rs519982 and rs7389) passed correction for multiple testing. All three SNPs were in high LD with each other (0.94 < r^2^ < 0.97), indicating that they represent a single association signal, making functional predictions difficult. However, we can speculate based on the putative individual SNP functions. Rs519982 is located in a region predicted to contain a transcription factor binding motif 14.9kb upstream of the *TREH* start codon. Its predicted location in a transcription factor binding site proximal to the *TREH* gene boundary may have functional implications; rs7389 is located in the 3’ UTR of *PHLDB1* and is predicted to affect microRNA (miRNA) binding site activity that can inhibit protein translation[59].

Our second most significant association, rs6713972, located in *pleckstrin homology domain containing family B member 2* (*PLEKHB2*), is in the same family as *PHLDB1*. Deficiency in another member of the pleckstrin homology containing gene family, *pleckstrin homology-like domain*, *family A*, *member 1* (*PHLDA1*) has been shown to be protective against atherosclerosis through regulation of cholesterol efflux, apoptosis, and peroxiredoxin-1 expression in mice[60]. Additionally, similar to *PAI-1*, *TREH* is a stress response gene known to associate with susceptibility to Type 2 diabetes [61,62].

Median regression analyses revealed novel variants associated with *PAI-1* levels that would not have been detected with linear regression. While linear regression may be appropriate for studies with extremely large sample sizes, for studies with modest sample sizes, such as ours, the impact of performing “standard” analyses can be significant.

Extending our analyses to include upper quartile regression allowed us to gain additional knowledge about the differential impact of genetic variants in this clinically significant portion of the *PAI-1* distribution. Elevated *PAI-1* levels are associated with increased susceptibility to CVD and in some cases severity of disease [5,63–65]. Knowledge of genetic variation on *PAI-1* levels at the higher end of the distribution may aid in the development of targeted therapies that may not be relevant to the general population, but could have a significant impact on a subset of the population already at increased risk of CVD.

## Supporting Information

S1 Table Corresponding Standard Linear Regression Results for SNPs found to be significantly associated with Plasminogen Activator Inhibitor-1 (PAI-1) levels by Median Regression. (DOCX)

S2 Table Hardy-Weinberg Equilibrium Estimates and allele frequencies of SNPs significantly associated with Median Plasminogen Activator Inhibitor-1 (PAI-1) levels. (DOCX)

S3 Table Genotypic Distribution of SNPs significantly associated with Median Plasminogen Activator Inhibitor 1 (PAI-1) levels. (DOCX)

S4 Table Hardy-Weinberg Equilibrium Estimates and allele frequencies of SNPS significantly associated with the Upper Quartile of Plasminogen Activator Inhibitor-1 (PAI-1) Distribution. (DOCX)

S5 Table Genotypic Distribution of SNPs significantly associated with the Upper Quartile of the Plasminogen Activator Inhibitor 1 (PAI-1) Distribution. (DOCX)

S1 Fig Study Cohort STRUCTURE analysis results. HapMap populations are anchored at each corner of the triangle part. HapMap individuals are represented by black dots; current study participants are represented by orange dots. An individual’s proportion of African ancestry decreases linearly with increasing distance from the top of the triangle (labeled YRI) which corresponds to 100% African ancestry.
(TIF)

## References

[1] Global status report on non-communicable diseases 2010. World Health Organization; 2011 [cited 2014]; Available: www.who.int.

[2] Rona G. The pathogenesis of human myocardial infarction. Canadian Medical Association journal. 1966;95(20):1012–9. 5924947 PMC1935795

[3] Booth NA, Bennett B. Fibrinolysis and thrombosis. Bailliere's clinical haematology. 1994;7(3):559–72. 7841601 10.1016/s0950-3536(05)80099-x

[4] Kawasaki T, Dewerchin M, Lijnen HR, Vermylen J, Hoylaerts MF. Vascular release of plasminogen activator inhibitor-1 impairs fibrinolysis during acute arterial thrombosis in mice. Blood. 2000;96(1):153–60. 10891445

[5] Juhan-Vague I, Morange PE, Frere C, Aillaud MF, Alessi MC, Hawe E, et al The plasminogen activator inhibitor-1–675 4G/5G genotype influences the risk of myocardial infarction associated with elevated plasma proinsulin and insulin concentrations in men from Europe: the HIFMECH study. J Thromb Haemost. 2003;1(11):2322–9. 14629464 10.1046/j.1538-7836.2003.00458.x

[6] Ridker PM, Hennekens CH, Lindpaintner K, Stampfer MJ, Miletich JP. Arterial and venous thrombosis is not associated with the 4G/5G polymorphism in the promoter of the plasminogen activator inhibitor gene in a large cohort of US men. Circulation. 1997;95(1):59–62. 8994417 10.1161/01.cir.95.1.59

[7] Cesari M, Sartori MT, Patrassi GM, Vettore S, Rossi GP. Determinants of plasma levels of plasminogen activator inhibitor-1: A study of normotensive twins. Arterioscler Thromb Vasc Biol. 1999;19(2):316–20. 9974413 10.1161/01.atv.19.2.316

[8] de Lange M, Snieder H, Ariens RA, Spector TD, Grant PJ. The genetics of haemostasis: a twin study. Lancet. 2001;357(9250):101–5. 11197396 10.1016/S0140-6736(00)03541-8

[9] Peetz D, Victor A, Adams P, Erbes H, Hafner G, Lackner KJ, et al Genetic and environmental influences on the fibrinolytic system: a twin study. Thrombosis and haemostasis. 2004;92(2):344–51. 15269831 10.1160/TH04-01-0001

[10] Asselbergs FW, Pattin K, Snieder H, Hillege HL, van Gilst WH, Moore JH. Genetic architecture of tissue-type plasminogen activator and plasminogen activator inhibitor-1. Seminars in thrombosis and hemostasis. 2008;34(6):562–8. 10.1055/s-0028-1103367 19085655

[11] Huang J, Sabater-Lleal M, Asselbergs FW, Tregouet D, Shin SY, Ding J, et al Genome-wide association study for circulating levels of PAI-1 provides novel insights into its regulation. Blood. 2012;120(24):4873–81. 10.1182/blood-2012-06-436188 22990020 PMC3520624

[12] Penrod NM, Poku KA, Vaughan DE, Asselbergs FW, Brown NJ, Moore JH, et al Epistatic interactions in genetic regulation of t-PA and PAI-1 levels in a Ghanaian population. PloS one. 2011;6(1):e16639 Epub 2011/02/10. 10.1371/journal.pone.0016639 21304999 PMC3031598

[13] Schoenhard JA, Asselbergs FW, Poku KA, Stocki SA, Gordon S, Vaughan DE, et al Male-female differences in the genetic regulation of t-PA and PAI-1 levels in a Ghanaian population. Human genetics. 2008;124(5):479–88. Epub 2008/10/28. 10.1007/s00439-008-0573-x 18953568 PMC2770717

[14] Iso H, Folsom AR, Koike KA, Sato S, Wu KK, Shimamoto T, et al Antigens of tissue plasminogen activator and plasminogen activator inhibitor 1: correlates in nonsmoking Japanese and Caucasian men and women. Thrombosis and haemostasis. 1993;70(3):475–80. Epub 1993/09/01. 8259552

[15] Lutsey PL, Cushman M, Steffen LM, Green D, Barr RG, Herrington D, et al Plasma hemostatic factors and endothelial markers in four racial/ethnic groups: the MESA study. Journal of thrombosis and haemostasis: JTH. 2006;4(12):2629–35. Epub 2006/09/28. 17002663 10.1111/j.1538-7836.2006.02237.x

[16] Festa A, D'Agostino R Jr., Rich SS, Jenny NS, Tracy RP, Haffner SM. Promoter (4G/5G) plasminogen activator inhibitor-1 genotype and plasminogen activator inhibitor-1 levels in blacks, Hispanics, and non-Hispanic whites: the Insulin Resistance Atherosclerosis Study. Circulation. 2003;107(19):2422–7. Epub 2003/04/30. 12719278 10.1161/01.CIR.0000066908.82782.3A

[17] Matthews KA, Sowers MF, Derby CA, Stein E, Miracle-McMahill H, Crawford SL, et al Ethnic differences in cardiovascular risk factor burden among middle-aged women: Study of Women's Health Across the Nation (SWAN). American heart journal. 2005;149(6):1066–73. Epub 2005/06/25. 15976790 10.1016/j.ahj.2004.08.027

[18] Koenker R, B G. Regression quantiles. Econometrica 1978 p. 33–50.

[19] Briollais L, Durrieu G. Application of quantile regression to recent genetic and -omic studies. Hum Genet. 2014. 10.1007/s00439-014-1440-6 24770874

[20] Koenker R. Quantile Regression. New York: Cambridge University Press; 2005.

[21] John O, N E. Quantile Regression Analysis as a robust alternative to ordinary least squares. Scientia Africana. 2009;8(2):61–5.

[22] Williams PT. Quantile-specific penetrance of genes affecting lipoproteins, adiposity and height. PloS one. 2012;7(1):e28764 10.1371/journal.pone.0028764 22235250 PMC3250394

[23] Williams SM, Stocki S, Jiang L, Brew K, Gordon S, Vaughan DE, et al A population-based study in Ghana to investigate inter-individual variation in plasma t-PA and PAI-1. Ethn Dis. 2007;17(3):492–7. 17985503

[24] Purcell S, Neale B, Todd-Brown K, Thomas L, Ferreira MA, Bender D, et al PLINK: a tool set for whole-genome association and population-based linkage analyses. American journal of human genetics. 2007;81(3):559–75. 17701901 10.1086/519795 PMC1950838

[25] StataCorp. Stata Statistical Software: Release 11. College Station, TX: StataCorp LP; 2009.

[26] Pritchard JK, Stephens M, Donnelly P. Inference of population structure using multilocus genotype data. Genetics. 2000;155(2):945–59. Epub 2000/06/03. 10835412 10.1093/genetics/155.2.945 PMC1461096

[27] Team RC. R: A Language and Environment for Statistical Computing. Vienna, Austria: R Foundation for Statistical Computing; 2013.

[28] Braun-Fahrlander C, Wuthrich B, Gassner M, Grize L, Sennhauser FH, Varonier HS, et al Validation of a rhinitis symptom questionnaire (ISAAC core questions) in a population of Swiss school children visiting the school health services. SCARPOL-team. Swiss Study on Childhood Allergy and Respiratory Symptom with respect to Air Pollution and Climate. International Study of Asthma and Allergies in Childhood. Pediatr Allergy Immunol. 1997;8(2):75–82. Epub 1997/05/01. 9617776 10.1111/j.1399-3038.1997.tb00147.x

[29] Abboud N, Ghazouani L, Saidi S, Ben-Hadj-Khalifa S, Addad F, Almawi WY, et al Association of PAI-1 4G/5G and -844G/A gene polymorphisms and changes in PAI-1/tissue plasminogen activator levels in myocardial infarction: a case-control study. Genetic testing and molecular biomarkers. 2010;14(1):23–7. 10.1089/gtmb.2009.0039 19929406

[30] Juhan-Vague I, Alessi MC, Morange PE. Hypofibrinolysis and increased PAI-1 are linked to atherothrombosis via insulin resistance and obesity. Ann Med. 2000;32 Suppl 1:78–84. 11209987

[31] Barrett JC, Fry B, Maller J, Daly MJ. Haploview: analysis and visualization of LD and haplotype maps. Bioinformatics. 2005;21(2):263–5. 15297300 10.1093/bioinformatics/bth457

[32] Xu Z, Taylor JA. SNPinfo: integrating GWAS and candidate gene information into functional SNP selection for genetic association studies. Nucleic Acids Res. 2009;37(Web Server issue):W600–5. 10.1093/nar/gkp290 19417063 PMC2703930

[33] Sunyaev S, Ramensky V, Koch I, Lathe W 3rd, Kondrashov AS, Bork P. Prediction of deleterious human alleles. Human molecular genetics. 2001;10(6):591–7. Epub 2001/03/07. 11230178 10.1093/hmg/10.6.591

[34] Yue P, Melamud E, Moult J. SNPs3D: candidate gene and SNP selection for association studies. BMC Bioinformatics. 2006;7:166 16551372 10.1186/1471-2105-7-166 PMC1435944

[35] Kel AE, Gossling E, Reuter I, Cheremushkin E, Kel-Margoulis OV, Wingender E. MATCH: A tool for searching transcription factor binding sites in DNA sequences. Nucleic Acids Res. 2003;31(13):3576–9. 12824369 10.1093/nar/gkg585 PMC169193

[36] Cartegni L, Wang J, Zhu Z, Zhang MQ, Krainer AR. ESEfinder: A web resource to identify exonic splicing enhancers. Nucleic Acids Res. 2003;31(13):3568–71. 12824367 10.1093/nar/gkg616 PMC169022

[37] Munson M, Balasubramanian S, Fleming KG, Nagi AD, O'Brien R, Sturtevant JM, et al What makes a protein a protein? Hydrophobic core designs that specify stability and structural properties. Protein science: a publication of the Protein Society. 1996;5(8):1584–93. 8844848 10.1002/pro.5560050813 PMC2143493

[38] Bhattacharyya S, Tobacman JK. Molecular signature of kappa-carrageenan mimics chondroitin-4-sulfate and dermatan sulfate and enables interaction with arylsulfatase B. The Journal of nutritional biochemistry. 2012;23(9):1058–63. 10.1016/j.jnutbio.2011.05.012 22079206

[39] Bhattacharyya S, Tobacman JK. Hypoxia reduces arylsulfatase B activity and silencing arylsulfatase B replicates and mediates the effects of hypoxia. PloS one. 2012;7(3):e33250 10.1371/journal.pone.0033250 22428001 PMC3302843

[40] Sorensen BS, Toustrup K, Horsman MR, Overgaard J, Alsner J. Identifying pH independent hypoxia induced genes in human squamous cell carcinomas in vitro. Acta oncologica. 2010;49(7):895–905. 10.3109/02841861003614343 20429727

[41] Wende H, Volz A, Ziegler A. Extensive gene duplications and a large inversion characterize the human leukocyte receptor cluster. Immunogenetics. 2000;51(8–9):703–13. 10941842 10.1007/s002510000187

[42] Normant E, Gros C, Schwartz JC. Carboxypeptidase A isoforms produced by distinct genes or alternative splicing in brain and other extrapancreatic tissues. J Biol Chem. 1995;270(35):20543–9. 7657630 10.1074/jbc.270.35.20543

[43] Bentley L, Nakabayashi K, Monk D, Beechey C, Peters J, Birjandi Z, et al The imprinted region on human chromosome 7q32 extends to the carboxypeptidase A gene cluster: an imprinted candidate for Silver-Russell syndrome. Journal of medical genetics. 2003;40(4):249–56. 12676894 10.1136/jmg.40.4.249 PMC1735416

[44] Pereira HJ, Souza LL, Costa-Neto CM, Salgado MC, Oliveira EB. Carboxypeptidases A1 and A2 from the perfusate of rat mesenteric arterial bed differentially process angiotensin peptides. Peptides. 2012;33(1):67–76. 10.1016/j.peptides.2011.12.001 22178042

[45] Ocaranza MP, Jalil JE. Protective Role of the ACE2/Ang-(1–9) Axis in Cardiovascular Remodeling. International journal of hypertension. 2012;2012:594361 10.1155/2012/594361 22315665 PMC3270559

[46] Skurk T, Lee YM, Rohrig K, Hauner H. Effect of angiotensin peptides on PAI-1 expression and production in human adipocytes. Horm Metab Res. 2001;33(4):196–200. 11383921 10.1055/s-2001-14948

[47] Brown NJ, Agirbasli M, Vaughan DE. Comparative effect of angiotensin-converting enzyme inhibition and angiotensin II type 1 receptor antagonism on plasma fibrinolytic balance in humans. Hypertension. 1999;34(2):285–90. 10454455 10.1161/01.hyp.34.2.285

[48] Asselbergs FW, Williams SM, Hebert PR, Coffey CS, Hillege HL, Navis G, et al Epistatic effects of polymorphisms in genes from the renin-angiotensin, bradykinin, and fibrinolytic systems on plasma t-PA and PAI-1 levels. Genomics. 2007;89(3):362–9. Epub 2007/01/09. 17207964 10.1016/j.ygeno.2006.11.004 PMC1808222

[49] Philippe C, Porter DE, Emerton ME, Wells DE, Simpson AH, Monaco AP. Mutation screening of the EXT1 and EXT2 genes in patients with hereditary multiple exostoses. American journal of human genetics. 1997;61(3):520–8. 9326317 10.1086/515505 PMC1715939

[50] Liu L, Yang X, Wang H, Cui G, Xu Y, Wang P, et al Association between variants of EXT2 and type 2 diabetes: a replication and meta-analysis. Hum Genet. 2013;132(2):139–45. 10.1007/s00439-012-1231-x 23052945

[51] Konkle BA, Kollros PR, Kelly MD. Heparin-binding growth factor-1 modulation of plasminogen activator inhibitor-1 expression. Interaction with cAMP and protein kinase C-mediated pathways. J Biol Chem. 1990;265(35):21867–73. 1701436

[52] Guess J, Burch JB, Ogoussan K, Armstead CA, Zhang H, Wagner S, et al Circadian disruption, Per3, and human cytokine secretion. Integrative cancer therapies. 2009;8(4):329–36. 10.1177/1534735409352029 19926609 PMC2959170

[53] Cheng B, Anea CB, Yao L, Chen F, Patel V, Merloiu A, et al Tissue-intrinsic dysfunction of circadian clock confers transplant arteriosclerosis. Proc Natl Acad Sci U S A. 2011;108(41):17147–52. 10.1073/pnas.1112998108 21969583 PMC3193243

[54] Ko CH, Takahashi JS. Molecular components of the mammalian circadian clock. Hum Mol Genet. 2006;15 Spec No 2:R271–7. 16987893 10.1093/hmg/ddl207

[55] Chong NW, Codd V, Chan D, Samani NJ. Circadian clock genes cause activation of the human PAI-1 gene promoter with 4G/5G allelic preference. FEBS Lett. 2006;580(18):4469–72. 16857194 10.1016/j.febslet.2006.07.014

[56] Schoenhard JA, Smith LH, Painter CA, Eren M, Johnson CH, Vaughan DE. Regulation of the PAI-1 promoter by circadian clock components: differential activation by BMAL1 and BMAL2. J Mol Cell Cardiol. 2003;35(5):473–81. 12738229 10.1016/s0022-2828(03)00051-8

[57] Stow LR, Gumz ML. The circadian clock in the kidney. J Am Soc Nephrol. 2011;22(4):598–604. 10.1681/ASN.2010080803 21436284 PMC5797999

[58] Ciarleglio CM, Ryckman KK, Servick SV, Hida A, Robbins S, Wells N, et al Genetic differences in human circadian clock genes among worldwide populations. Journal of biological rhythms. 2008;23(4):330–40. 10.1177/0748730408320284 18663240 PMC2579796

[59] Valencia-Sanchez MA, Liu J, Hannon GJ, Parker R. Control of translation and mRNA degradation by miRNAs and siRNAs. Genes & development. 2006;20(5):515–24. 16510870 10.1101/gad.1399806

[60] Hossain GS, Lynn EG, Maclean KN, Zhou J, Dickhout JG, Lhotak S, et al Deficiency of TDAG51 protects against atherosclerosis by modulating apoptosis, cholesterol efflux, and peroxiredoxin-1 expression. Journal of the American Heart Association. 2013;2(3):e000134 10.1161/JAHA.113.000134 23686369 PMC3698773

[61] Muller YL, Hanson RL, Knowler WC, Fleming J, Goswami J, Huang K, et al Identification of genetic variation that determines human trehalase activity and its association with type 2 diabetes. Hum Genet. 2013;132(6):697–707. 10.1007/s00439-013-1278-3 23468175 PMC3654185

[62] Ouyang Y, Xu Q, Mitsui K, Motizuki M, Xu Z. Human trehalase is a stress responsive protein in Saccharomyces cerevisiae. Biochem Biophys Res Commun. 2009;379(2):621–5. 10.1016/j.bbrc.2008.12.134 19126402

[63] Iwai N, Shimoike H, Nakamura Y, Tamaki S, Kinoshita M. The 4G/5G polymorphism of the plasminogen activator inhibitor gene is associated with the time course of progression to acute coronary syndromes. Atherosclerosis. 1998;136(1):109–14. 9544737 10.1016/s0021-9150(97)00191-3

[64] Alessi MC, Juhan-Vague I. Contribution of PAI-1 in cardiovascular pathology. Archives des maladies du coeur et des vaisseaux. 2004;97(6):673–8. 15283042

[65] Aso Y. Plasminogen activator inhibitor (PAI)-1 in vascular inflammation and thrombosis. Frontiers in bioscience: a journal and virtual library. 2007;12:2957–66. 17485272 10.2741/2285

