# Supplementary material for: Genetics of Plasminogen Activator Inhibitor-1 (PAI-1) in a Ghanaian Population
Source: PLoS One. 2015 Aug 31;10(8):e0136379. doi: 10.1371/journal.pone.0136379 (PMC4556460; doi:10.1371/journal.pone.0136379)
Supplement: S1 Table — (DOCX) [file pone.0136379.s001.docx]

**S1 Table. Corresponding Standard Linear Regression Results for SNPs found to be significantly associated** **with Plasminogen Activator Inhibitor-1 (PAI-1) levels by Median Regression**

| **Chr.** | **Gene** | **SNP** | **Beta^a.^** | **SE^b.^** | **95% Confidence Interval** | | **P-value** |
| --- | --- | --- | --- | --- | --- | --- | --- |
|  |  |  |  |  | **LL** | **UL** |  |
| 4 | *SLC7A11* | rs4479754 | 0.233 | 0.062 | -0.356 | -0.111 | 2.28E-04 |
| 5 | *ARSB* | rs1071598 | -0.429 | 0.142 | -0.708 | -0.151 | 0.003 |
| 7 | *CPA2* | rs61997065 | 0.376 | 0.137 | 0.107 | 0.645 | 0.006 |
| 19 | *LENG9* | rs10406453 | -0.253 | 0.095 | -0.434 | -0.065 | 0.008 |
| 19 | *LENG8* | rs1035451 | -0.326 | 0.107 | -0.535 | -0.117 | 0.002 |
| 7 | *SERPINE1* | rs1799768 (PAI-1 4G/5G) | 0.247 | 0.065 | 0.120 | 0.375 | 1.51E-04 |

Results that remained significant after FDR correction are highlighted in **bold**.

^a.^Beta coefficient from median regression model represents the effect of the minor allele; model covariates: age, sex, BMI, triglycerides, and PAI1 4G/5G variant genotype.

^b.^SE; Standard error; robust standard errors are reported above.

LL= 95% Confidence Interval lower limit; UL= 95% Confidence Interval upper limit
