# Supplementary material for: Genetics of Plasminogen Activator Inhibitor-1 (PAI-1) in a Ghanaian Population
Source: PLoS One. 2015 Aug 31;10(8):e0136379. doi: 10.1371/journal.pone.0136379 (PMC4556460; doi:10.1371/journal.pone.0136379)
Supplement: S2 Table — (DOCX) [file pone.0136379.s002.docx]

**S2 Table. Hardy-Weinberg Equilibrium Estimates and allele frequencies of SNPs significantly associated with Median Plasminogen Activator Inhibitor-1 (PAI-1) levels**

| **Chr.** | **Gene** | **SNP** | **Minor Allele** | **Major Allele** | **MAF^a.^** | **HWE**  **P-value^b.^** |
| --- | --- | --- | --- | --- | --- | --- |
| 5 | *ARSB* | rs1071598 | T | C | 0.048 | 0.726 |
| 7 | *CPA2* | rs61997065 | A | G | 0.045 | 0.466 |
| 19 | *LENG9* | rs10406453 | T | C | 0.075 | 0.652 |

^a.^ MAF; Minor Allele Frequency

^b.^HWE P-value; Hardy-Weinberg Equilibrium P-value
