# Supplementary material for: Genetics of Plasminogen Activator Inhibitor-1 (PAI-1) in a Ghanaian Population
Source: PLoS One. 2015 Aug 31;10(8):e0136379. doi: 10.1371/journal.pone.0136379 (PMC4556460; doi:10.1371/journal.pone.0136379)
Supplement: S3 Table — (DOCX) [file pone.0136379.s003.docx]

**S3 Table. Genotypic Distribution of SNPs significantly associated with Median Plasminogen Activator Inhibitor 1 (PAI-1) levels**

| **Chr.** | **Gene** | **SNP^a.^** | **Minor Allele** | **Major Allele** | **Genotype**  **Distribution^b.^** | | |
| --- | --- | --- | --- | --- | --- | --- | --- |
|  |  |  |  |  | ***mm*** | ***Mm*** | ***MM*** |
| 5 | *ARSB* | rs1071598 | T | C | 1 | 98 | 954 |
|  |  | *rs1071598_dom* |  |  | 99 | | 954 |
| 7 | *CPA2* | rs61997065 | A | G | 3 | 89 | 961 |
|  |  | *rs61997065_dom* |  |  | 92 | | 961 |
| 19 | *LENG9* | rs10406453 | T | C | 7 | 143 | 902 |

^a.^Instances in which sample size was below 5 for any genoptype group, SNPs were recoded dominantly for the effect of the minor allele (homozygous minor and heterozygotes were combined) prior to regression analyses; *_dom* denotes dominant coding genotype distribution

^b.^*mm* = homozygous minor, *Mm* = heterozygote, *MM* = homozygous major
