# Supplementary material for: Genetics of Plasminogen Activator Inhibitor-1 (PAI-1) in a Ghanaian Population
Source: PLoS One. 2015 Aug 31;10(8):e0136379. doi: 10.1371/journal.pone.0136379 (PMC4556460; doi:10.1371/journal.pone.0136379)
Supplement: S4 Table — (DOCX) [file pone.0136379.s004.docx]

**S4 Table. Hardy-Weinberg Equilibrium Estimates and allele frequencies of SNPS significantly associated with the Upper Quartile of Plasminogen Activator Inhibitor-1 (PAI-1) Distribution**

| **Chr.** | **Gene** | **SNP** | **Minor Allele** | **Major Allele** | **MAF^a.^** | **HWE**  **P-value^b.^** |
| --- | --- | --- | --- | --- | --- | --- |
| 1 | *COL16A1* | rs72887331 | A | C | 0.141 | 0.610 |
| 1 | *FHAD1* | rs12126178 | A | G | 0.131 | 0.177 |
| 1 | *PER3* | rs10462021 | G | A | 0.070 | 0.473 |
| 2 | *PLECKHB2* | rs6713972 | G | T | 0.088 | 0.029 |
| 3 | *--* | rs13314993 | T | G | 0.077 | 0.272 |
| 3 | *SLC15A2* | rs116307792 | G | A | 0.054 | 1.000 |
| 5 | *ADAMTS12* | rs61757473 | C | G | 0.049 | 0.509 |
| 6 | *TAGAP* | rs35263580 | T | C | 0.053 | 0.113 |
| 7 | *--* | rs2023783 | A | G | 0.070 | 0.475 |
| 9 | *DBH* | rs4531 | T | G | 0.146 | 0.901 |
| 11 | *EXT2* | rs4755779 | G | A | 0.071 | 0.231 |
| 11 | *PHLDB1 / TREH* | rs7389 | C | A | 0.232 | 0.339 |
|  | *TREH* | rs519982 | T | C | 0.230 | 0.163 |
| 12 | *OR1OP1* | rs76940436 | T | A | 0.065 | 0.441 |
| 12 | *P2RX7* | rs34219304 | A | G | 0.050 | 0.177 |
| 14 | *NID2* | rs2273430 | C | A | 0.248 | 0.868 |
| 14 | *FAM161B* | rs34834232 | T | A | 0.114 | 0.359 |
| 16 | *C1QTNF8* | rs73494080 | G | T | 0.051 | 0.105 |
| 17 | *CEP95* | rs9910506 | A | G | 0.055 | 1.000 |

a.MAF; Minor Allele Frequency

^b.^HWE P-value; Hardy-Weinberg Equilibrium P-value
