# Supplementary material for: Genetics of Plasminogen Activator Inhibitor-1 (PAI-1) in a Ghanaian Population
Source: PLoS One. 2015 Aug 31;10(8):e0136379. doi: 10.1371/journal.pone.0136379 (PMC4556460; doi:10.1371/journal.pone.0136379)
Supplement: S5 Table — (DOCX) [file pone.0136379.s005.docx]

**S5 Table. Genotypic Distribution of SNPs significantly associated with the Upper Quartile of the Plasminogen Activator Inhibitor 1 (PAI-1) Distribution**

| **Chr.** | **Gene** | **SNP^a.^** | **Minor Allele** | **Major Allele** | **Genotype Distribution^b.^** | | |
| --- | --- | --- | --- | --- | --- | --- | --- |
|  |  |  |  |  | ***mm*** | ***Mm*** | ***MM*** |
| 1 | *COL16A1* | rs72887331 | A | C | 23 | 251 | 779 |
| 1 | *FHAD1* | rs12126178 | A | G | 23 | 230 | 800 |
| 1 | *PER3* | rs10462021 | G | A | 3 | 141 | 909 |
|  |  | rs10462021*_dom* |  |  | 144 | | 909 |
| 2 | *PLECKHB2* | rs6713972 | G | T | 14 | 153 | 867 |
| 3 | *--* | rs13314993 | T | G | 9 | 144 | 900 |
| 3 | *SLC15A2* | rs116307792 | G | A | 3 | 108 | 942 |
| 5 | *ADAMTS12* | rs61757473 |  |  |  |  |  |
| 6 | *TAGAP* | rs35263580 | T | C | 6 | 100 | 944 |
|  |  | rs35263580*_dom* |  |  | 106 | | 944 |
| 7 | *--* | rs2023783 | A | G | 3 | 142 | 908 |
|  |  | rs2023783*_dom* |  |  | 145 | | 908 |
| 9 | *DBH* | rs4531 | T | G | 23 | 261 | 769 |
| 11 | *EXT2* | rs4755779 | G | A | 8 | 132 | 913 |
| 11 | *PHLDB1 / TREH* | rs7389 | C | A | 50 | 384 | 610 |
|  | *TREH* | rs519982 | T | C | 47 | 390 | 616 |
| 12 | *OR1OP1* | rs76940436 | T | A | 6 | 125 | 922 |
| 12 | *P2RX7* | rs34219304 | A | G | 5 | 95 | 952 |
| 14 | *FAM161B* | rs34834232 | T | A | 17 | 207 | 829 |
| 14 | *NID2* | rs2273430 | C | A | 65 | 387 | 591 |
| 16 | *C1QTNF8* | rs73494080 | G | T | 0 | 107 | 946 |
|  |  | rs73494080*_dom* |  |  | 107 | | 946 |
| 17 | *CEP95* | rs9910506 | A | G | 3 | 110 | 940 |
|  |  | rs9910506*_dom* |  |  | 113 | | 940 |
| 20 | *DEFB132* | rs74420259 | A | G | 3 | 112 | 937 |
|  |  | rs74420259*_dom* |  |  | 115 | | 937 |

^a.^Instances in which sample size was below 5 for any genoptype group, SNPs were recoded dominantly for the effect of the minor allele (homozygous minor and heterozygotes were combined) prior to regression analyses; *_dom* denotes dominant coding genotype distribution

^b.^*mm* = homozygous minor, *Mm* = heterozygote, *MM* = homozygous major
