# Supplementary figures and images for: Genetics of Plasminogen Activator Inhibitor-1 (PAI-1) in a Ghanaian Population
Source: PLoS One. 2015 Aug 31;10(8):e0136379. doi: 10.1371/journal.pone.0136379 (PMC4556460; doi:10.1371/journal.pone.0136379)

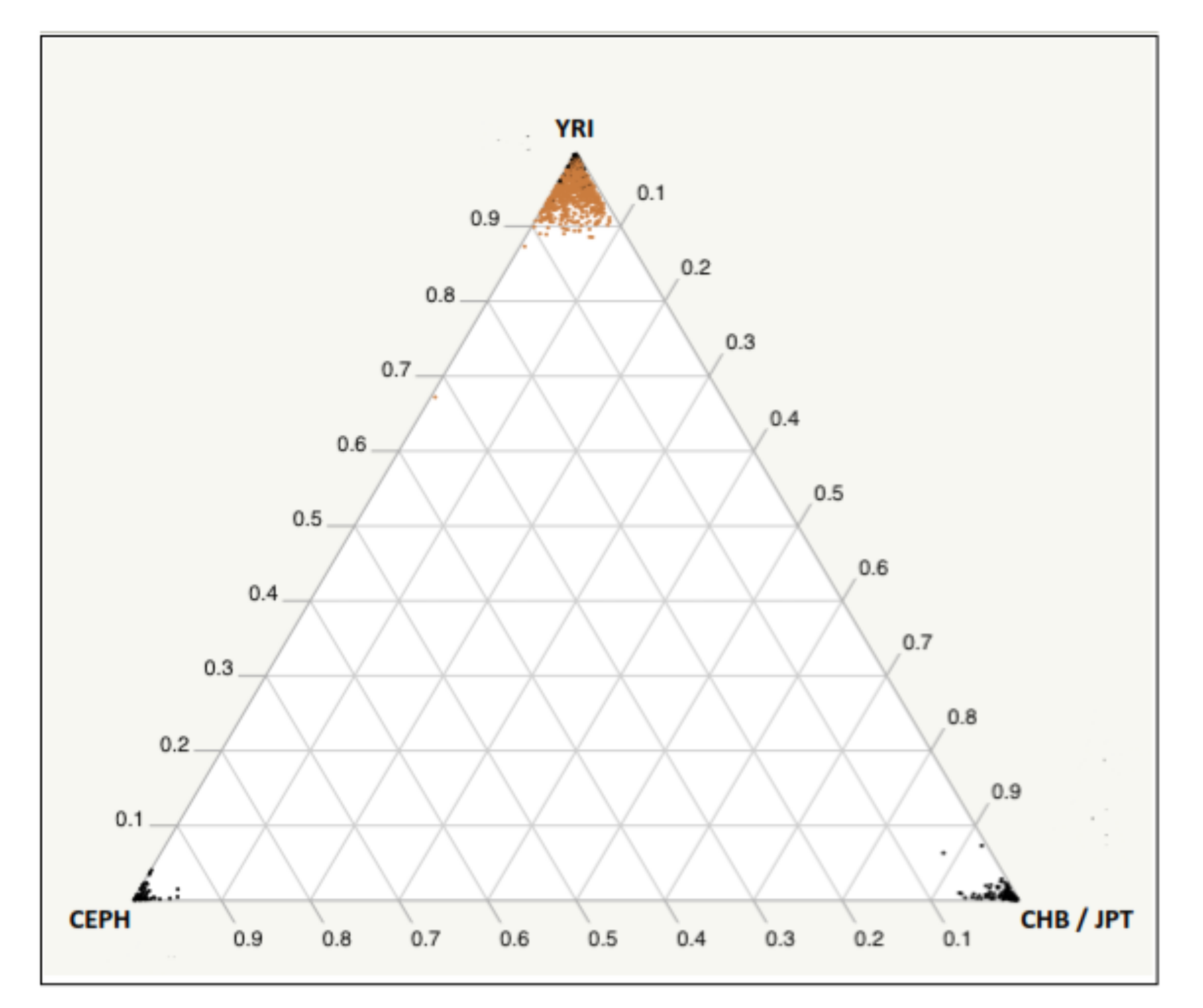

Supplement: S1 Fig — HapMap populations are anchored at each corner of the triangle part. HapMap individuals are represented by black dots; current study participants are represented by orange dots. An individual’s proportion of African ancestry decreases linearly with increasing distance from the top of the triangle (labeled YRI) which corresponds to 100% African ancestry. (TIF) [file pone.0136379.s006.tif]
